# Identification of an at-risk subpopulation with high immune infiltration based on the peroxisome pathway and TIM3 in colorectal cancer

**DOI:** 10.1186/s12885-021-09085-9

**Published:** 2022-01-07

**Authors:** Jinwen Yin, Hao Wang, Yuntian Hong, Anli Ren, Haizhou Wang, Lan Liu, Qiu Zhao

**Affiliations:** 1 Department of Gastroenterology, Zhongnan Hospital of Wuhan University, Wuhan, 430000 China; 2 Hubei Clinical Center and Key Lab of Intestinal and Colorectal Diseases, Wuhan, 430000 China; 3 Department of Colorectal and Anal Surgery, Zhongnan Hospital of Wuhan University, Wuhan, 430000 China

**Keywords:** Colorectal cancer (CRC), Peroxisome, T-cell immunoglobulin and mucin domain 3 (TIM-3), Immunotherapy, Gene set variant analysis (GSVA), Fatty acid alpha oxidation (FAAO)

## Abstract

**Background:**

Peroxisomes are pivotal metabolic organelles that exist in almost all eukaryote cells. A reduction in numbers and enzymatic activities of peroxisomes was found in colon adenocarcinomas. However, the role of peroxisomes or the peroxisome pathway in colorectal cancer (CRC) is not defined.

**Methods:**

In the current study, a peroxisome score was calculated to indicate the activity of the peroxisome pathway using gene set variant analysis based on transcriptomic datasets. CIBERSORTx was chosen to infer enriched immune cells for tumors among subgroups. The SubMap algorithm was applied to predict its sensitivity to immunotherapy.

**Results:**

The patients with a relatively low peroxisome score and high level of T-cell immunoglobulin and mucin domain 3 (TIM-3) presented the worse overall survival than others. Moreover, low peroxisome scores were associated with high infiltration of lymphocytes and poor prognosis in those CRC patients. Thus, a PER^Low^TIM3^High^ CRC risk subpopulation was identified and characterized by high immune infiltration. The results also showed that CD8 T cells and macrophages highly infiltrated tumors of the PER^Low^TIM3^High^ group, regardless of consortium molecular subtype and microsatellite instability status. This subgroup had the highest tumor mutational burden and overexpression of immune checkpoint genes. Further, the PER^Low^TIM3^High^ group showed a higher probability of responding to programmed cell death protein-1-based immunotherapy. In addition, genes involved in peroxisomal metabolic processes in CRC were also investigated since peroxisome is a rather pleiotropic and highly metabolic organelle in cell. The results indicated that only those genes involved in fatty acid alpha oxidation could be used to stratify CRC patients as similar as peroxisome pathway genes.

**Conclusions:**

We revealed the favorable prognostic value of the peroxisome pathway in CRC and provided a new CRC stratification based on peroxisomes and TIM3, which might be helpful for CRC diagnostics and personalized treatment.

**Supplementary Information:**

The online version contains supplementary material available at 10.1186/s12885-021-09085-9.

## Background

Colorectal cancer (CRC), one of the most prevalent malignancies, is the second leading cause of cancer-related death worldwide [1]. Although the use of chemotherapy and targeted therapy has dramatically prolonged survival time for patients with unresectable CRCs, the efficacy of these therapies can be limited by drug resistance and side effects [2]. In the past few years, a better knowledge of the complex interactions between tumor cells and the immune system has led to the establishment of novel immunotherapies [3, 4]. However, only a small subset of metastatic CRCs with the microsatellite instability (MSI) phenotype benefit from immune checkpoint inhibitors (ICIs) that target co-inhibitory receptors [5, 6]. One major immunotherapeutic obstacle of CRC is the immunosuppressive tumor microenvironment (TME), a highly complex and heterogenous contexture [7, 8]. For CRC tumors with pre-existing strong immune infiltration, restoring the suppressive effects of the TME on immune cells is a potential strategy to improve the efficacy of immunotherapy [9]. Thus, TME-based classifications are valuable for personalized immunotherapy in CRC.

Peroxisomes are membrane-bound organelles first discovered by Johannes Rhodin in 1954. Peroxisomes are involved in multiple metabolic processes, including the metabolism of fatty acid oxidation and D-amino acids, biosynthesis of ether lipids and bile acids, and turnover of reactive oxygen species (ROS) [10]. Apart from being a highly metabolic organelle, the peroxisome has emerged as a pivotal regulator of inflammation and immune responses in these few years [11–13]. Furthermore, a recent study suggested that enhanced peroxisome signaling might be involved in reprograming the lipid metabolism of T cells in urological cancers [14]. The attention on the role of peroxisomes or peroxisomal lipid metabolism in cancer, a disease characterized by metabolic reprogramming, is continuously increasing. From earlier publications, an overall reduction in peroxisomal proteins and enzymatic activities has been found in neoplastic tissue, including that of the colon [15–17]. Moreover, a more recent study suggested that NUDT7, a peroxisomal gene, is a potent tumor suppressor during KRAS^G12D^-driven CRC development [18]. Although these publications pointed to a potential loss of peroxisome function during CRC tumorigenesis, our current view on the role of peroxisomes or the peroxisome pathway in CRC remains fragmentary and limited.

In the current study, the prognostic role of peroxisome pathway was evaluated in CRC based on the genomic data of public datasets. In addition, an at-risk CRC subpopulation was identified and characterized by massive immune infiltration. Then, we explored the relationship between the newly characterized subpopulation and immunotherapy efficiency. Finally, we further investigated whether the genes specifically involved in peroxisomal metabolic processes can stratify CRC patients individually.

## Methods

### Data collection and processing

This study used two public patient datasets for discovery and validation. The CRC cohort from the Cancer Genome Atlas (TCGA) was used for discovery. We downloaded the RNA-Seq expression data (FPKM values) of 593 primary colon or rectum tumors with overall survival (OS) data using the *GDCquery* of the R package *TCGAbiolinks* [19]*.* We then transformed the FPKM values into transcripts per kilobase million (TPM) values for subsequent analysis. We used a consensus measurement of tumor purity to remove samples with low tumor purity (< 60%) [20]. We updated the clinical information from TCGA Pan-Cancer Clinical Data Resource [21]. A total of 504 TCGA CRC patients enrolled in this study possessed gene somatic mutation data (MAF files). We analyzed and summarized the mutation data using *maftools* [22]*.* A cohort from the Gene Expression Omnibus (GEO), GSE39582 (*n* = 556), was used for validation. We downloaded the normalized expression data and clinical information for the GSE39582 dataset from the GEO repository. We obtained the MSI status and consortium molecular subtype (CMS) classifications from the Colorectal Cancer Subtyping Consortium synapse data portal (https://www.synapse.org/#!Synapse:syn2623706/files/). We further combined MSI-low and microsatellite stable (MSS) cases and defined them as MSS and defined MSI-high cases as MSI for this analysis.

### Gene set expression analyses

Gene set variant analysis (GSVA) is a computational approach that has been utilized to measure the overall activity of biological pathways [23–25]. With a pathway-based approach, the expression of pathway member genes can be summarized thus reducing complexity, and yielding more reliable results [26]. We applied it to the transcriptomic data samples to estimate the HALLMARK_PEROXISOME score from the Molecular Signatures Database (MSigDB) Hallmark collection using the *GSVA* R package (version 3.10) [23]. The median value of the GSVA HALLMARK_PEROXISOME score was used as the cut-off to categorize the CRC patients into either a peroxisome-high (Per-High) or peroxisome-low (Per-Low) group. We used the gene set enrichment analysis (GSEA) method to investigate the relationship between HALLMARK_PEROXISOME and other Hallmark gene sets using the *GSEABase* package (http://www.bioconductor.org/packages/release/bioc/html/GSEABase.html) [27]. Gene set related to β-oxidation of very-long-chain fatty acids was obtained from Reactome Pathway database (https://reactome.org/) [28]. Gene sets associated with fatty acid alpha oxidation and ROS were from Gene Ontology (http://geneontology.org/) [29, 30]. Gene sets related to biosynthesis of bile acids and ether lipid metabolism were from Kyoto Encyclopedia of Genes and Genomes (KEGG) database (https://www.kegg.jp/kegg/kegg1.html) [31, 32].

### Estimation of immune infiltration

The approach utilized for estimating immune infiltration was CIBERSORTx (https://cibersortx.stanford.edu/), which can predict the abundance and proportion of 22 types of tumor-infiltrating lymphocytes based on the expression of 547 genes for each tissue sample [33]. We additionally applied the ESTIMATE (Estimation of STromal and Immune cells in MAlignant Tumours using Expression data) algorithm to assess the enrichment of immune and stromal cells in the TME and to infer purity [34].

### Prediction of the immunotherapy response for each subgroup

To predict the sensitivity of each subgroup to immunotherapy, we used Class mapping analysis (SubMap from Gene Pattern, https://www.genepattern.org/) to compare the similarity of the gene expression profiles of our subgroups to those of melanoma patients treated with checkpoint blockades against programmed cell death protein-1 (PD1) or cytotoxic T lymphocyte antigen-4 (CTLA4) [35].

### Statistical analysis

We applied the optimal cut-off for T-cell immunoglobulin and mucin domain 3 (TIM3) expression with regard to the associated hazard of death events in a *survfit* model based on the *cutp* function of the *survMisc* R package (version 0.5.5; https://CRAN.R-project.org/package=survMisc). We chose the highest log-rank test score as the optimal cut-off for classifying patients into high- and low-expression groups with different risks. Kaplan–Meier analysis was used to estimate OS. We conducted multivariate Cox proportional hazards regression models to investigate the prognostic value of group III using the *survival* R package (version 2.42.3; https://CRAN.R-project.org/package=survival). To compare the contingency table variables, we used the chi-square test or Fisher’s exact test. We used the Mann–Whitney test for two-group comparison and the Kruskal–Wallis test for three-group comparisons. All statistical results with a *p*-value < 0.05 were considered significant.

## Results

### A low peroxisome pathway score is associated with a worse clinical outcome and high immune cell infiltration in CRC patients

**Fig. 1:**
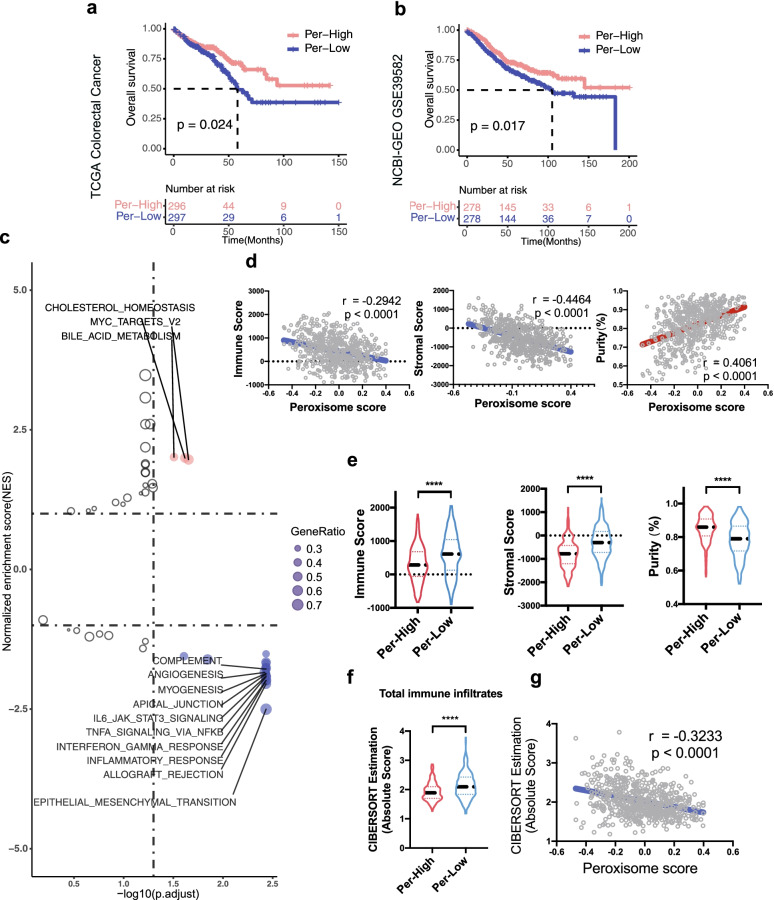
Peroxisome-Low CRC patients had poor clinical outcomes despite high immune infiltrates. **a**, **b** Kaplan–Meier analysis of OS in TCGA colorectal cancer cohort (*n* = 593) and NCBI GEO-GSE39582 cohort (*n* = 556). *P* values were calculated by the log-rank test. **c** The volcano plot shows the enrichment analysis using hallmark gene sets in TCGA cohort. The red gene sets were enriched in the Per-High group, whereas the blue gene sets were enriched in the Per-Low group. **d** Correlation between the peroxisome score versus immune score, stromal score, and tumor purity in TCGA cohort. **e** Violin plots of the immune score, stromal score, and tumor purity from ESTIMATE of Per-High and Per-Low groups in TCGA cohort. **f** Violin plot of total immune infiltrates (sum of absolute scores across 22 immune cell types) among the CRC subgroups in TCGA. **g** Correlation between peroxisome scores and total immune infiltrates of patients in TCGA cohort. For violin plots, *p* values in group comparisons with Mann–Whitney U–test are shown. For panels d, Pearson’s rho (r) and statistical difference (p) are indicated. ****P < 0.0001

The peroxisome score was calculated by GSVA for CRC patients enrolled in the study and patients were categorized into either a Per-High or Per-Low group by the median of peroxisome scores. Intriguingly, the Per-Low group showed significantly worse OS than the Per-High group in TCGA CRC dataset (*p* = 0.024; Fig. 1a), findings that were validated in the GSE39582 dataset (*p* = 0.017; Fig. 1b). We next performed GSEA on 50 hallmark gene sets in TCGA cohort and found lipid-associated gene sets, CHOLESTEROL_HOMEOSTASIS and BILE_ACID_METABOLISM, which were significantly enriched in the Per-High group (Fig. 1c), which was validated in the GSE39582 cohort (Additional file 2: Fig. S1a). Furthermore, we found that immune-related gene sets were highly enriched in the Per-Low group, both in TCGA discovery dataset and the GEO-GSE39582 validation dataset (Fig. 1c; Additional file 2: Fig. S1a; Additional file 1: Table S1–2).

To further investigate the association between the peroxisome pathway and TME in CRC, we applied the ESTIMATE algorithm to TCGA cohort. We observed that the peroxisome pathway activity was negatively correlated with immune and stromal cell infiltration but was positively correlated with tumor purity (Fig. 1d). The Per-Low group had significantly higher immune and stromal scores but a lower level of tumor purity than the Per-High group (Fig. 1e). We also used a deconvolution method, CIBERSORTx, to assess the total immune infiltrates. The Per-Low group demonstrated higher immune cell infiltration than the Per-High group (Fig. 1f). A negative correlation between peroxisome scores and total immune infiltrates was observed (Fig. 1g). These results were validated with the GSE39582 dataset (Additional file 2: Fig. S1b-d). Taken together, our findings revealed that patients with low peroxisome scores had poor clinical outcomes despite having a high degree of immune infiltrates.

### Identification and validation of an at-risk CRC subpopulation stratified by peroxisome score and TIM3 expression

**Fig. 2:**
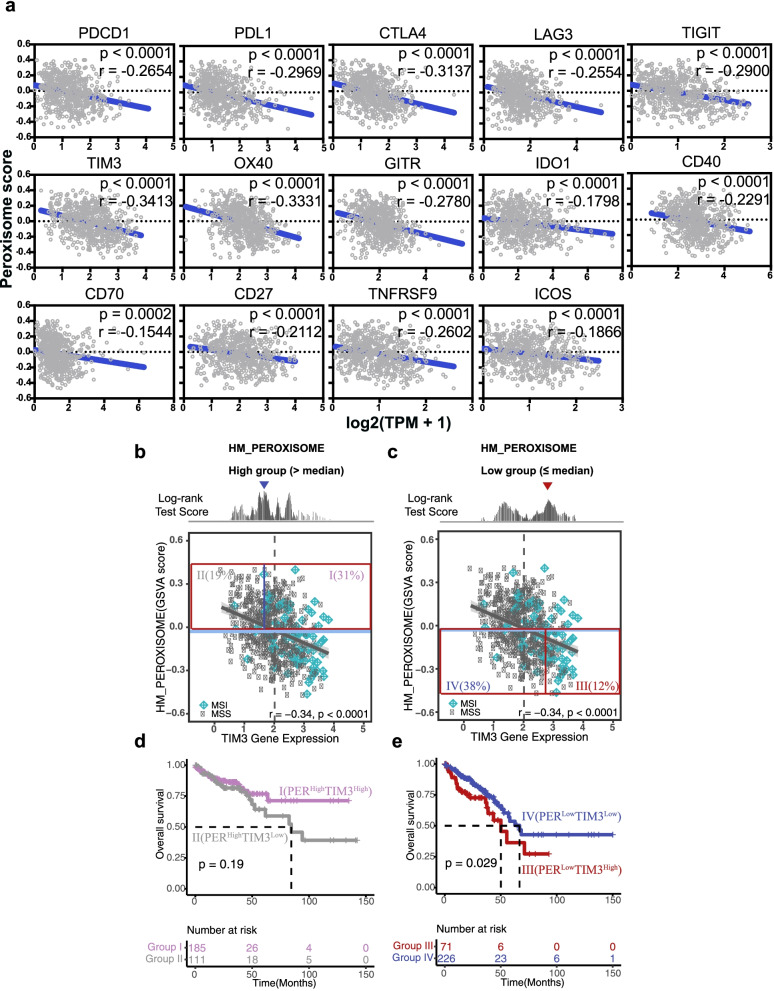
Identification of an at-risk CRC subpopulation stratified by peroxisome score and TIM3 expression. **a** Pairwise correlations of the peroxisome score versus expression of individual immune checkpoint genes in TCGA cohort. Pearson’s rho (r) and statistical difference (p) are shown in each graph. **b, c** Scatter plots of peroxisome score and log2-transformed TIM3 gene expression values are shown for TCGA dataset. MSI (blue diamond) and MSS (black circle) status are labeled for CRC patients. The median value of TIM3 expression is indicated in a gray dashed line. The log-rank test score patterns and candidate cut-offs (blue and red arrows) for high peroxisome and low peroxisome groups are shown in panels **b** and **c**, respectively. **d**, **e** Kaplan–Meier curves show the OS for the optimal cut-off of TIM3 expression in TCGA CRC subgroups with high peroxisome score and low peroxisome score, respectively. Using the median value of peroxisome score and these two candidate cut-offs, we separated patients into four different groups labeled with their population fraction in percentage (group I-IV). Kaplan–Meier curves are plotted for the risk groups in high peroxisome and low peroxisome groups, respectively. *P*-value was calculated by the log-rank test and shown for each plot

**Fig. 3:**
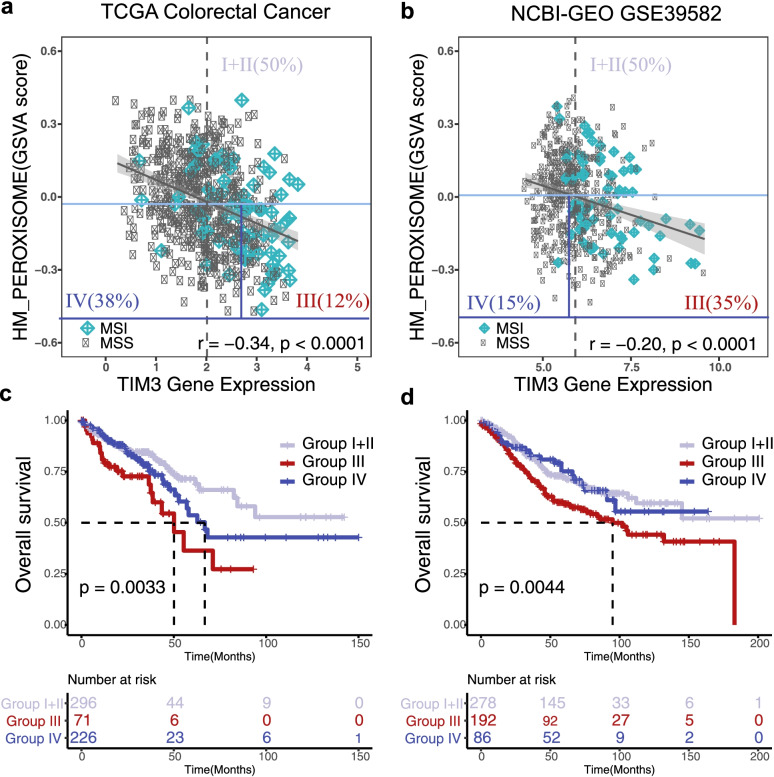
Validation of the risk CRC population using NCBI-GEO GSE39582 dataset. **a**, **b** Scatter plots of peroxisome score and log2-transformed TIM3 gene expression values are shown for TCGA and NCBI-GEO GSE39582 cohort. Three CRC subgroups were indicated for each plot (group I + II: high peroxisome group; group III: PER^Low^TIM3^High^ group; group IV: PER^Low^TIM3^Low^ group). **c**, **d** For OS analysis, Kaplan–Meier curves are plotted for the risk subgroups in TCGA and GSE39582 datasets. The log-rank p values are shown

To further investigate immunosuppressive mechanisms that could contribute to the poor prognosis observed in the Per-Low group, we analyzed the expression of immune checkpoints (ICs), as these genes are associated with the failure of T cells to efficiently eliminate tumor cells. The Per-Low group showed higher levels of all inhibitory checkpoint genes that we examined (Additional file 2: Fig. S2), including *PDCD1* (*PD1*), *PDL1*, *CTLA4*, *LAG3*, *TIGIT*, *TIM3*, *OX40*, *GITR*, *TNFRSF9* (*4-1BB*), *ICOS*, *IDO1*, *CD40*, *CD70*, and *CD27*. We observed that the peroxisome score was negatively correlated with the respective expression levels of these checkpoint genes in TCGA cohort (Fig. 2a). Among these ICs, TIM3 had the highest negative correlation with the peroxisome score. Additionally, we used an optimal cut-off for TIM3 expression to classify CRC patients into high and low TIM3 groups, followed by OS analysis. We found that for TCGA colorectal tumors, a high level of TIM3 expression was associated with a poor prognosis (Additional file 2: Fig. S3), which is consistent with the findings of previous studies [36, 37]. Intriguingly, Figs. 2b-e show that TIM3 prognostic behaviors were dependent on peroxisome scores. The median peroxisome score was used to classify patients into the Per-High and Per-Low groups. We applied an optimal cut-off based on the log-rank test for each independent group and subsequently defined four groups as follows: group I (PER^High^TIM3^High^), group II (PER^High^TIM3^Low^), group III (PER^Low^TIM3^High^), and group IV (PER^Low^TIM3^Low^) (Fig. 2b and c). In the Per-Low group, we found that TIM3 served as a prognostic marker of poor outcome; however, this was not seen in the Per-High group (Fig. 2d and e). Group III (PER^Low^TIM3^High^), with the highest proportion of MSI-status tumors, showed the most unfavorable outcomes compared with those in group IV and the Per-High group (group I + II), based on an analysis of OS (Fig. 3a and c). In addition, we adopted the same method to validate the existence of risk group III in the GSE39582 validation dataset. Group III consistently had the worst OS, as expected (Fig. 3b and d). Our results showed that group III patients had the worst OS; therefore, it would be of interest to explore differences in clinical and molecular characteristics across three subpopulations.

We gathered clinical and molecular data of 1149 CRC patients from TCGA and GSE39582. Characteristics of all patients are shown in Table 1. Advanced age and stages, a higher mutation frequency of BRAF, a CpG-island methylator phenotype (CIMP)-high status, and the MSI phenotype were associated with group III. Furthermore, we investigated the proportions of different CMS subtypes across subgroups and found that group III largely overlapped with CMS1 (MSI immune subtype; strong immune infiltration and activation) and CMS4 (mesenchymal; TGF-β activation; stromal invasion and angiogenesis).

**Table 1:** Clinical and molecular characteristics of the CRC subgroups stratified by peroxisome score and TIM3 expression

	***n*** = 1149	Group I + II (Peroxisome^**High**^) ***n*** = 574 (50%)	Group III (Per^**Low**^TIM3^**High**^) ***n*** = 263 (23%)	Group IV (Per^**Low**^TIM3^**Low**^) ***n*** = 312 (27%)	p
**Age (years)**					
< 65	445	239 (54%)	86 (19%)	120 (27%)	
≥ 65	704	335 (48%)	177 (25%)	192 (27%)	**0.048**
**Gender**					
Female	526	261 (49%)	119 (23%)	146 (28%)	
Male	623	313 (50%)	144 (23%)	166 (27%)	0.913
**Stage**					
I	133	74 (56%)	18 (14%)	41 (31%)	
II	474	253 (53%)	110 (23%)	111 (23%)	
III	371	169 (46%	94 (25%)	108 (29%)	
IV	147	65 (44%)	37 (25%)	45 (31%)	**0.020**
**Location**					
Left colon, rectum	549	277 (50%)	114 (21%)	158 (29%)	
Right colon	578	146 (25%)	146 (25%)	286 (50%)	0.149
**Microsatellite**					
MSI	150	64 (43%)	66 (44%)	20 (13%)	
MSS	949	480 (51%)	178 (19%)	291 (31%)	**< 0.001**
**CMS**					
CMS1	160	65 (41%)	77 (48%)	18 (11%)	
CMS2	445	274 (62%)	42 (9%)	129 (29%)	
CMS3	136	89 (65%)	10 (7%)	37 (27%)	
CMS4	253	74 (29%)	106 (42%)	73 (29%)	
Indeterminate	106	54 (51%)	24 (23%)	28 (26%)	**< 0.001**
**KRAS status**					
Mutated	374	192 (51%)	92 (25%)	90 (24%)	
Wild-type	664	314 (47%)	156 (23%)	194 (29%)	0.197
**BRAF status**					
Mutated	102	36 (35%)	48 (47%)	18 (18%)	
Wild-type	903	455 (50%)	192 (21%)	256 (28%)	**< 0.001**
**TP53 status**					
Mutated	496	222 (45%)	97 (20%)	177 (36%)	
Wild-type	351	191 (54%)	76 (22%)	84 (24%)	**0.001**
**CIMP**					
High	164	64 (39%)	70 (43%)	30 (18%)	
Low	771	420 (54%)	159 (21%)	192 (25%)	**< 0.001**

To assess the independent poor prognostic value of group III, we performed univariate and multivariate cox regression analysis to investigate the association between traditional clinical and molecular characteristics and OS for the entire cohort. We found three independent indicators for poor OS, stage IV (Stage IV: HR = 6.07, 95% CI: 3.25–11.35, *p* < 0.001; Stage I as the reference), mutated KRAS (HR = 1.29, 95% CI: 1.00–1.66, *p* = 0.048) and group III (Group III: HR = 1.41, 95% CI: 1.05–1.90, *p* = 0.023; Group I + II as the reference). Whereas group III (PER^Low^TIM3^High^) had the highest proportions of CMS1 and CMS4 subtypes, of interest, our multivariable OS model showed that group III, but not CMS4, was an independent indicator for predicting poor OS (Table 2).

**Table 2:** Univariate and multivariate analyses for overall survival in CRC

	Univariate analysis		Multivariate analysis	
	HR (95% CI)	p	HR (95% CI)	p
**Gender**				
Female	1			
Male	1.23 (0.98–1.50)	0.076		
**Stage**				
I	1		1	
II	1.70 (0.94–3.10)	0.082	1.45 (0.79–2.66)	0.225
III	2.34 (1.28–4.25)	0.006	1.74 (0.95–3.21)	0.074
IV	8.26 (4.50–15.18)	< 0.001	6.07 (3.25–11.35)	**< 0.001**
**Location**				
Left colon, rectum	1			
Right colon	1.13 (0.90–1.42)	0.304		
**Microsatellite**				
MSI	1			
MSS	1.11 (0.78–1.58)	0.566		
**CMS**				
CMS1	1		1	
CMS2	0.97 (0.67–1.38)	0.848	0.94 (0.63–1.39)	0.743
CMS3	0.70 (0.42–1.16)	0.171	0.67 (0.37–1.17)	0.160
CMS4	1.45 (1.00–2.11)	0.049	1.10 (0.74–1.64)	0.625
Indeterminate	1.55 (0.98–2.46)	0.064	1.41 (0.87–2.29)	0.159
**KRAS status**				
Wild-type	1		1	
Mutated	1.27 (1.01–1.61)	0.043	1.29 (1.00–1.66)	**0.048**
**BRAF status**				
Wild-type	1			
Mutated	1.23 (0.85–1.79)	0.28		
**TP53 status**				
Wild-type	1			
Mutated	1.20 (0.92–1.57)			
**CIMP**				
Low	1			
High	1.14 (0.83–1.57)			
**Group**				
Group I + II	1		1	
Group III	1.68 (1.30–2.17)	< 0.001	1.41 (1.05–1.90)	**0.023**
Group IV	1.20 (0.90–1.60)	0.223	1.06 (0.77–1.48)	0.709

GSE39582 and TCGA samples of CRC were pooled for this analysis

*Abbreviations*: *HR* hazard ratio

### The PER^Low^TIM3^High^ group has high levels of tumor infiltrated CD8+ T cells and macrophages

**Fig. 4:**
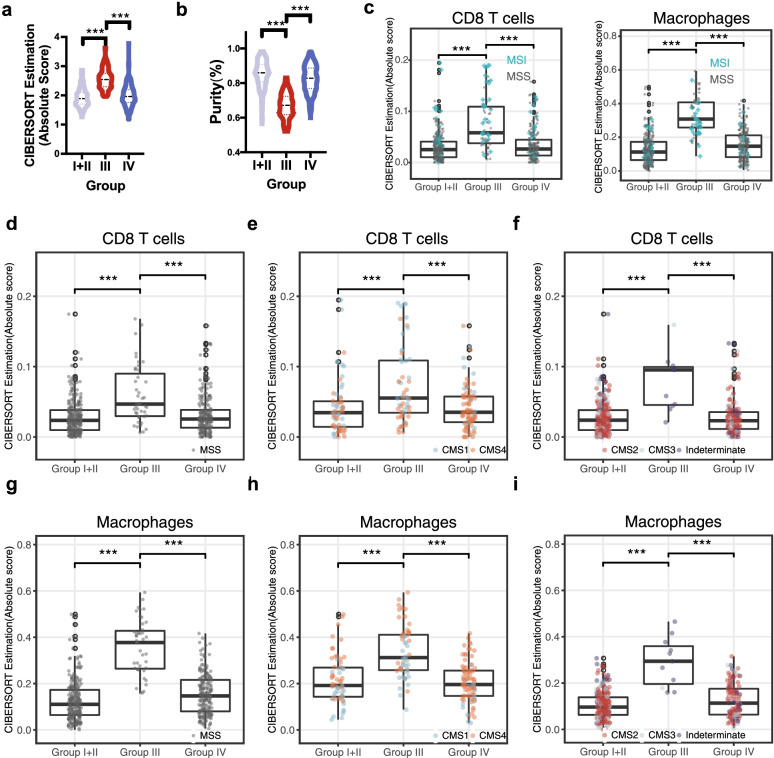
TCGA group III (PER^Low^TIM3^High^) tumors were highly infiltrated with CD8 T cells and macrophages. **a** Violin plot showing the total immune infiltrates of CIBERSORTx for each subgroup in TCGA CRC dataset. **b** Violin plot showing the ESTIMATE tumor purity for each subgroup in TCGA CRC dataset. **c** Boxplots showing enrichment levels of CD8 T cells and macrophages for each subgroup in TCGA. **d**, **g** Boxplots of enrichment level of CD8 T cells and macrophages for TCGA MSS tumors. **e**, **h** Boxplots of enrichment level of CD8 T cells and macrophages for TCGA CMS1 and CMS4 tumors. **f**, **i** Boxplots of enrichment level of CD8 T cells and macrophages for TCGA CMS2, CMS3, and indeterminate tumors. ***P < 0.001

To explore the immune heterogeneity across subgroups, we used CIBERSORTx to estimate total immune infiltrates. Group III had the highest enrichment level of immune-infiltrated cells (Fig. 4a; Additional file 2: Fig. S4a). We then used the ESTIMATE-based approach to infer tumor purity; compared with group I + II and IV, group III had the lowest tumor purity (Fig. 4b; Additional file 2: Fig. S4b).

To further investigate the proportions of immune infiltrates in group III, we aggregated CIBERSORTx results of relative cell-types into six major cell types (B cells, CD4 T cells, CD8 T cells, dendritic cells, macrophages, and neutrophils). We observed that CD8 T cells and macrophages were highly enriched in group III both in TCGA and GSE39582 datasets (Fig. 4c; Additional file 2: Fig. S4c). CRC MSS tumors tend to have poor immunogenicity and lack responses to ICIs [38]; our results showed that group III MSS tumors were also highly infiltrated by CD8 T cells and macrophages (Fig. 4d and g; Additional file 2: Fig. S4d and S4g). As mentioned, group III had high proportions of CMS1 and CMS4, and these types were related to high immune infiltration. Next, we compared tumor infiltrating levels of CD8 T cells and macrophages between CMS1 + CMS4 patients among the three subgroups; CMS1 + CMS4 tumors in group III had the highest enrichment levels (Fig. 4e and h; Additional file 2: Fig. S4e and S4h). Moreover, other CMS subtypes (CMS2, CMS3, and indeterminate) tumors in group III had the highest infiltration of CD8 T cells and macrophages among the three subgroups (Fig. 4f and i; Additional file 2: Fig. S4f and S4i). Together, these results indicate that group III tumors were highly infiltrated by CD8 T cells and macrophages, which was not a simple reflection of high proportions of MSI tumors or CMS1/CMS4 phenotypes.

### The PER^Low^TIM3^High^ group has the highest tumor mutational burden (TMB) and is likely to respond to immunotherapy

**Fig. 5:**
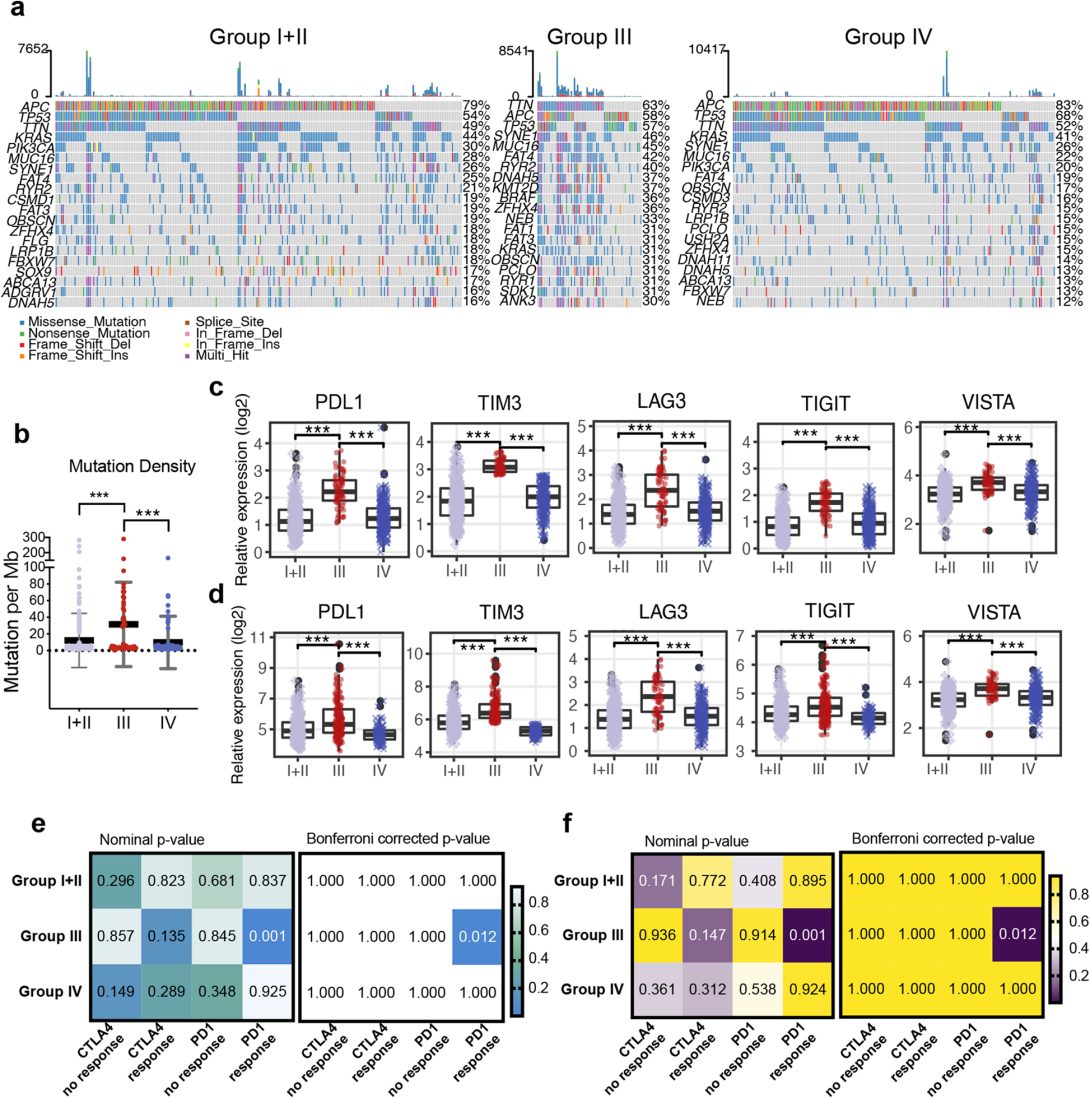
Comparison of tumor mutational burden and possible sensitivity to immunotherapy for CRC subgroups. **a** Top 20 genes most frequently mutated in TCGA CRC patients in groups I + II, III, and IV. **b** Comparison of the tumor mutation load across the three CRC subgroups in TCGA. **c** Plots show immune checkpoint genes of the three CRC subgroups in TCGA. **d** The same as panel **c**, but for the GSE39582 cohort. **e** Heatmaps show the correlation between transcriptomic expression patterns of melanoma patients receiving PD1 or CTLA4 inhibitors and CRC subgroups in TCGA. **f** The same as panel **e**, but for the GSE39582 dataset. ***P < 0.001

To further explore whether these groups have different mutational profiles, we analyzed somatic mutation data from TCGA. Figure 5a shows the 20 most frequently mutated genes for each subgroup. We observed that the mutational load increased significantly in this group (Fig. 5b).

Given that group III was associated with the highest immune infiltration level and TMB, we next sought to assess the possibility of efficient immunotherapy for the three subgroups. We found that group III had the highest expression of ICs in both TCGA and GSE39582 cohorts (Fig. 5c-d), including *PDL1*, *TIM3*, *LAG3*, *TIGIT*, and *VISTA*. We then used subclass mapping to measure the similarity of expression profiles between the three CRC subgroups and a public cohort for melanoma patients treated with checkpoint blockades against CTLA4 or PD-1 [35]. When comparing the expression profiles of the TCGA CRC subgroups and this external melanoma cohort, we observed that group III had a significant correlation with the PD-1 response group (Bonferroni corrected *p* = 0.012; Fig. 5e), a finding that was validated in the GSE39582 dataset (Bonferroni corrected p = 0.012; Fig. 5f). These results suggested that group III patients were likely to benefit from immunotherapy and specifically PD1-based strategies.

### Genes involved in fatty acid α-oxidation stratified CRC patients similarly with the peroxisome pathway

**Fig. 6:**
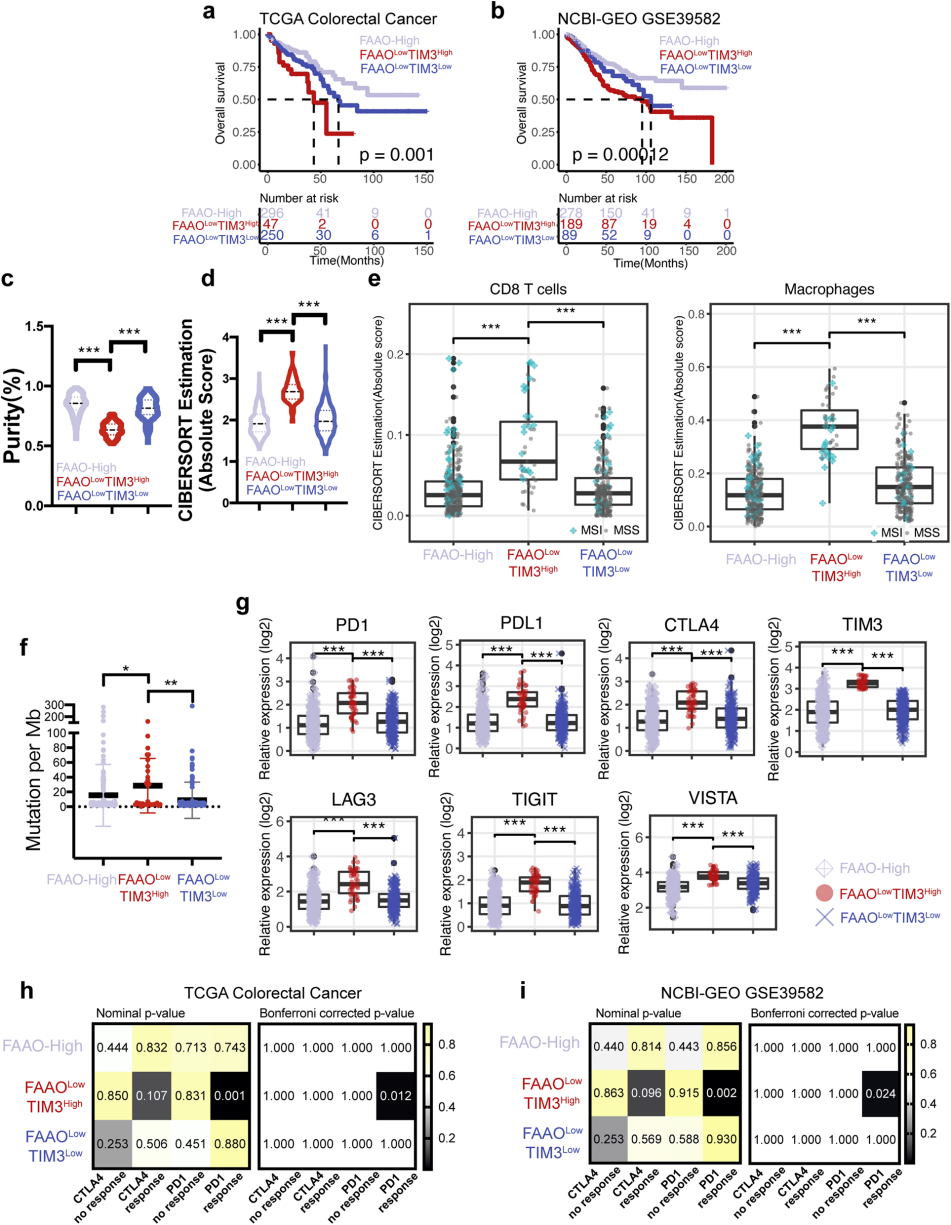
Genes involved in FAAO can stratify CRC patients similarly with genes in the peroxisome pathway. **a** Kaplan–Meier curves are plotted for the risk subgroups based on FAAO score and TIM3 expression in TCGA. **b** The same as **a**, but for the GSE39582 dataset. **c**, **d** Violin plots showing the ESTIMATE tumor purity (c) and the enrichment level of total immune infiltrates (d) for each subgroup based on FAAO stratification in TCGA. **e** Boxplots showing enrichment levels of CD8 T cells and macrophages for the three subgroups based on FAAO stratification in TCGA. **f**, **g** Comparisons of the tumor mutation load (f) and expression of immune checkpoint genes (g) across the three subgroups stratified by FAAO score and TIM3 expression in TCGA. **h**, **i** Heatmaps show the correlation between transcriptomic expression patterns of melanoma patients receiving PD1 or CTLA4 inhibitors and CRC subgroups stratified by FAAO score and TIM3 in TCGA (h) and GSE39582 (i). *P < 0.05; **P < 0.01; ***P < 0.001

Since peroxisomes are highly dynamic and pleiotropic metabolic organelles, we further investigated whether the genes specifically involved in peroxisomal metabolic processes can stratify CRC patients individually. Thus, we attempted to stratify TCGA CRC patients by using gene sets associated with specific peroxisome metabolic functions including REACTOME_BETA_OXIDATION_OF_VERY_LONG_CHAIN_FATTY_ACIDS (BOVLCFA), GOBP_FATTY_ACID_ALPHA_OXIDATION (FAAO), KEGG_PRIMARY_BILE_ACID_BIOSYNTHESIS (PBAB), KEGG_ETHER_LIPID_METABOLISM (ELM) and GOBP_CELLULAR_RESPONSE_TO_REACTIVE_OXYGEN_SPECIES (CRTROS) (Additional file 2: Fig. S5). For TCGA CRC patients with a relatively low BOVLCFA score or FAAO score, high TIM3 expression indicated worse OS (Additional file 2: Fig. S5a and S5b). However, our multivariable OS models showed that FAAO^Low^TIM3^High^, but not BOVLCFA^Low^TIM3^High^, was an independent predictor for poor OS (Additional file 1: Table S3–4). Thus, among these five gene sets, FAAO was found as the only gene set that could stratify CRC patients similarly as HALLMARK_PEROXISOME (Additional file 2: Fig. S5b); TCGA CRC patients with a relatively low FAAO score and high TIM3 expression (FAAO^Low^TIM3^High^) had the worst OS, which was validated in GSE39582 (Fig. 6a and b). Similarly, FAAO^Low^TIM3^High^ group tumors had the lowest tumor purity and were highly infiltrated by immune cells, especially CD8 T cells and macrophages (Fig. 6c-e). We also observed that in TCGA, FAAO^Low^TIM3^High^ group tumors had significantly increased TMB and expression of ICs (Fig. 6f and g). Then, we applied the SubMap to compare the expression profiles of TCGA CRC subgroups stratified by FAAO and TIM3 with the external melanoma cohort mentioned previously herein. We found that the FAAO^Low^TIM3^High^ group had a significant correlation with the PD-1 response group, which was validated in GSE39582 (Fig. 6h and i). Taken together, these results showed that genes involved in FAAO could be used to stratify CRC patients as similar as peroxisome pathway genes; moreover, tumors in the PER^Low^TIM3^High^ group and the FAAO^Low^TIM3^High^ groups displayed some similar characteristics.

## Discussion

Although there is a continuously increasing interest in peroxisomes and their functions in health and disease, the prognostic value of the peroxisome pathway has not been investigated. Our work first revealed a prognostic role of the peroxisome pathway in CRC, based on resources of genomic data from public clinical datasets. Then, we identified an at-risk CRC subpopulation based on the peroxisome pathway and TIM3 expression, and patients in this group might exhibit an improved response to immunotherapy. This new stratification was further refined by looking at the specific peroxisome functions, such as beta oxidation of very long-chain fatty acids, FAAO, biosynthesis of bile acids and ether lipids, and ROS turnover. Unexpectedly, we found that only genes involved in FAAO can stratify patients similarly with genes in the peroxisome pathway.

Peroxisomes are critical metabolic organelles associated with lipid metabolism and cellular ROS turnover [10]. Cablé and colleagues found reduced numbers of peroxisomes in colon carcinoma by electron microscopy [16]. However, the role of the peroxisome pathway in colorectal cancer has not been determined. As multiple peroxisomal biogenesis proteins and various oxidases are involved in peroxisome activity, we defined a peroxisome score to summarize the activity of the peroxisome pathway. We found that patients with a low peroxisome score had shorter OS than those with a high peroxisome score. Moreover, the score was associated with peroxisome-related pathways (e.g., the bile acid metabolism pathway and fatty acid metabolism pathway). Within the past few years, the idea that peroxisomes are vital organelles in regulating inflammation and the anti-microbial response has emerged [11–13, 39]. Vijayan and colleagues observed that deletion of the peroxisomal biogenesis factors PEX14 or MFP2 leads to a pronounced hyperexpression of COX2 and TNF-α proteins [13]. Deletion of PEX13, another peroxisomal biogenesis factor, causes the activation of Smad-dependent TGF-β signaling and the release of inflammatory cytokines, such as TGF-β and IL-6 [40]. Our results also showed that patients with a low peroxisome score had high immune infiltration and enriched immune-related pathways, despite the poor prognosis.

IC pathways are crucial in preventing autoimmunity and maintaining immune homeostasis; however, their upregulation in the TME of various malignancies can lead to T cell inactivation and immune evasion [41]. By blocking the interactions between ICs and their ligands, ICIs can efficiently initiate anti-tumor immune responses and have shown promise in eliminating tumor cells in some cancers. TIM3 (encoded by Havcr2), as one IC, is an inhibitory receptor expressed by various immune cells and was initially identified as a cell surface marker specific to CD4+ T helper 1 and CD8+ T cells [42, 43]. In cancer, the upregulation of TIM3 expression marks the most terminally exhausted CD8+ T cell subsets [44, 45]. Previous studies have shown that TIM3 expression in the tissues of CRC patients is associated with tumor progression and poor clinical outcomes [36, 37]. Our results showed that for tumors with a low peroxisome score, high TIM3 expression indicated poor OS. Intriguingly, even if it was not statistically significant, high TIM3 expression seemed to be correlated with a better prognosis for tumors with a high peroxisome score, suggesting the possible role of the peroxisome pathway in regulating biological behaviors of TIM3. Further investigations are needed to validate this hypothesis. Our genomic analysis revealed that the prognostic performance of TIM3 was dependent on the peroxisome score, and in agreement with this, we identified an at-risk CRC subpopulation (PER^Low^TIM3^High^) with a negative prognostic value.

Beyond the negative prognostic value of group III (PER^Low^TIM3^High^), an activated immune phenotype characterized by high levels of pro-tumorous and anti-tumorous immune cells was observed for these tumors. Tumor-infiltrating CD8+ T cells are the most potent tumor-suppressing immune cells. CD8+ T cell abundance was an independent predictor of better disease-free and OS outcomes in early CRC patients, leading to the establishment of the Immunoscore, a reliable independent prognostic marker for CRC [46, 47]. Our data show that tumors in group III had the highest CD8+ T cell abundance. Given that high TIM3 expression is associated with dysfunctional CD8+ T cells, it is not surprising that group III had a poor prognosis in the presence of high CD8+ T cell infiltration. Compared with tumors in other subgroups, group III tumors had the highest level of macrophages, as one of the important types of tumor-supportive immune cells. Tumor-associated macrophages, mostly in the type M2 form, are important for the inhibition of anti-tumor immune responses mediated by T cells within the TME [48, 49]. The risk subgroup largely overlapped with CMS1 and CMS4 subtypes, which are associated with high immune infiltration, but the enrichment of CD8 T cells and macrophages was not a simple reflection of the high proportion of these two CMS subtypes. Moreover, our multivariate cox regression model determined the independent prognostic value of group III for OS. Hence, the peroxisome/TIM3 stratification is distinct from the CMS classification of CRC [50]. We also found an association between group III tumors and MSI status. Our observation might promote refinement of the current clinical dogma that MSI-high tumors are associated with a good prognosis and encourage the further stratification of MSI tumors by peroxisome score and TIM3 expression [51, 52].

Consistent with the concept that MSI-high CRC is associated with high tumor mutations, group III tumors had the highest TMB. Moreover, high TMB was determined to be a predictive biomarker for immunotherapy in several tumor types [53, 54]. Immunotherapy is increasingly being recognized as a major treatment strategy for multiple cancers, including melanoma and a subset of CRC patients [55, 56]. PD1 blockade, as the only immunotherapeutic strategy approved by the FDA, has shown efficacy in metastatic CRC patients with MSI-high status [6, 57, 58]. Further, based on preclinical data, co-blockade of TIM3 and PD1 has shown efficacy for solid tumors, leading to the clinical investigation of anti-TIM3 combined with anti-PD1 in the treatment of various malignancies, including CRC [44, 59–61]. The sensitivity to immunotherapy is likely determined by many factors, such as the presence of CD8+ T cells, high TMB, and the expression of ICs [54, 62]. It is well established that metastatic CRC patients with an MSI-high status benefit from anti-PD1/PD-L1 therapy [63]. Our results suggest that patients in group III (enriched with MSI tumors) had poor clinical outcomes but are likely to benefit from treatment targeting PD1. As such, the peroxisome pathway might be an overlooked factor involved in predicting the response to immunotherapy. In addition to anti-PD1 therapy, the co-blockade of PD1 and TIM3 could prove to be a more suitable treatment strategy for group III patients. Although immunotherapy approaches have been unsuccessful for MSS tumors, highly immune-infiltrated MSS tumors in group III could be more likely to respond to ICIs than MSS tumors in other subgroups. Further clinical trials investigating the co-blockade of PD1 and TIM3 in selected group III patients might be warranted.

Since peroxisomes are pleiotropic organelles involved in multiple metabolic processes, the meaning of high peroxisome scores seems obscure. Thus, we also attempted to refine the stratification by genes involved in peroxisomal metabolic processes. We found that CRC patients can be stratified by genes only involved in FAAO, which was similarly with genes in the peroxisome pathway. FAAO is a specific peroxisomal metabolic process in which some unusual fatty acids, such as 2-hydroxy fatty acids and 3-methyl-branched fatty acids (i.e., phytanic acid), are shortened by one carbon atom, which is known to occur in peroxisomes [64, 65]. Peroxisomal FAAO is deficient in patients with peroxisome biogenesis disorders, which is reflected by the accumulation of phytanic acid (PA) in the plasma [66]. However, the role of FAAO or PA in CRC is not clear yet. Our results showed that only for CRC patients with a relatively low peroxisome score or FAAO score was high TIM3 expression indicative of poor OS, suggesting the possible role for normal peroxisomal FAAO in regulating biological functions of TIM3 in CRC, which is a new direction for our future work.

The retrospective nature of this analysis, conducted using public datasets, is the main limitation of our study. Furthermore, although we recapitulated the peroxisome pathway and identified the prognostic role of the peroxisome for CRC, assessing the correlation between the peroxisome score and the abundance of peroxisomes was outside the scope of our study. However, our findings suggested the value of the peroxisome pathway and the peroxisomal FAAO for CRC stratification, as well as the possible role of peroxisomes in TME and immunotherapy.

## Conclusions

Collectively, we evaluated the prognostic role of the peroxisome pathway in CRC and identified an at-risk CRC subpopulation (PER^Low^TIM3^High^), exhibiting high immune infiltration. Patients with PER^Low^TIM3^High^ tumors might more likely respond to anti-PD1 based immunotherapy. When looking at individual peroxisomal functions to refine the stratification, only genes involved in FAAO can stratify patients as similar as genes in the peroxisome pathway. Combined evaluation of the expression of TIM3 and genes involved in the peroxisome pathway or FAAO might be used for CRC diagnostics and could be helpful for personalized treatment.

## Supplementary Information

**Additional file 1: Table S1**: Pathway enrichment analysis of peroxisome-high and peroxisome-low groups in TCGA dataset. **Table S2**: Pathway enrichment analysis of peroxisome-high and peroxisome-low groups in GEO-GSE39582 dataset. **Table S3**. Univariate and multivariate analyses of the relationship between subgroups stratified by FAAO/TIM3 and clinical characteristics. **Table S4**. Univariate and multivariate analyses of the relationship between subgroups stratified by BOVLCFA/TIM3 and clinical characteristics.

**Additional file 2: Figure S1**. a The volcano plot shows the enrichment analysis using Hallmark gene sets in the GSE39582 cohort. The red gene sets were enriched in the Per-High group, whereas the blue gene sets were enriched in the Per-Low group. b Correlation between the peroxisome score versus immune score, stromal score, and tumor purity in the GSE39582 cohort. c Boxplots of the immune score, stromal score, and tumor purity from ESTIMATE of Per-High and Per-Low groups in the GSE39582 cohort. d Boxplot of total immune infiltrates (sum of absolute scores across 22 immune cell types) and correlation between peroxisome score and total immune infiltrates of patients in TCGA cohort. For Boxplots, *p* values in group comparison with Mann-Whitney U-test are shown. For panels B, Pearson’s rho (r) and statistical difference (p) are indicated. **P < 0.01; ****P < 0.0001. **Figure S2**. Boxplots depict the expression of immune checkpoint genes in the TCGA colorectal dataset. Statistical *P* values between groups were determined by Mann-Whitney U-test. HM_PEROXISOME: Hallmark Peroxisome gene set. **P < 0.01, ***P < 0.001, ****P < 0.0001. **Figure S3**. a The log-rank test score at the candidate cut-off across the log-transformed TIM3 gene expression values is plotted. b Kaplan-Meier curves are plotted for the TIM3-High group and TIM3-Low group by the optimal cut-off shown in panel a. **Figure S4**. GSE39582 group III tumors were highly infiltrated with CD8 T cells and macrophages. a Violin plot showing the total immune infiltrates of CIBERSORTx for each subgroup in the GSE39582 CRC dataset. b Violin plot showing the ESTIMATE tumor purity for each subgroup in the GSE39582 CRC dataset. c Boxplots showing enrichment levels of CD8 T cells and macrophages for each subgroup in the GSE39582 dataset. d, g Boxplots of enrichment level of CD8 T cells (d) and macrophages (g) MSS tumors. e, h Boxplots of enrichment level of CD8 T cells (e) and macrophages (h) for GSE39582 CMS1 and CMS4 tumors. f, i Boxplots of enrichment level of CD8 T cells (f) and macrophages (i) for GSE39582 CMS2, CMS3, and indeterminate tumors. *P < 0.05; **P < 0.01; ***P < 0.001. **Figure S5**. a Scatter plot of BOVLCFA score and log2-transformed TIM3 gene expression values are shown for TCGA cohort. The Pearson’s rho (r) and statistical difference (p) are shown in the scatter plot. MSI (blue diamond) and MSS (black circle) status are labeled for CRC patients. The median value of TIM3 expression is indicated in a gray dashed line. The candidate cut-offs (black lines) for high BOVLCFA and low BOVLCFA groups are shown. Using the median value of BOVLCFA score and these two candidate cut-offs, we separated patients into four different groups labeled. Kaplan-Meier curves show the OS for the optimal cut-off of TIM3 expression in TCGA CRC subgroups with high BOVLCFA score and low BOVLCFA score, respectively. *P*-value was calculated by the log-rank test and shown for each plot. b The same as panel a, but for FAAO score. c The same as panel a, but for PBAB score. d The same as panel a, but for ELM score. e The same as panel a, but for CRTROS score. BOVLCFA: beta oxidation of very long chain fatty acids; PBAB: primary bile acid biosynthesis; ELM: ether lipid metabolism; CRTROS: cellular response to reactive oxygen species.

## Data Availability

The data from our study were all openly available from the GEO database and TCGA repository (https://portal.gdc.cancer.gov/; https://www.ncbi.nlm.nih.gov/geo/query/acc.cgi).

## Abbreviations

CRC: Colorectal cancer
GSVA: Gene set variant analysis
TIM3: T-cell immunoglobulin and mucin domain 3
CMS: Consortium molecular subtype
MSI: Microsatellite instability
TMB: Tumor mutational burden
PD-1: Programmed cell death protein-1
FAAO: Fatty acid alpha oxidation
ICI: Immune checkpoint inhibitor
TME: Tumor microenvironment
ROS: Reactive oxygen species
TCGA: The Cancer Genome Atlas
TPM: Transcripts per kilobase million
GEO: Gene Expression Omnibus
MSS: Microsatellite stable
IC: Immune checkpoint
MSigDB: The Molecular Signatures Database
GSEA: Gene set enrichment analysis
KEGG: Kyoto Encyclopedia of Genes and Genomes
ESTIMATE: Estimation of STromal and Immune cells in MAlignant Tumours using Expression data
PD1: programmed cell death protein-1
CTLA4: cytotoxic T lymphocyte antigen–4
IC: immune checkpoint
CIMP: CpG-island methylator phenotype
BOVLCFA: beta oxidation of very long chain fatty acids
PBAB: Primary bile acid biosynthesis
ELM: Ether lipid metabolism
CRTROS: Cellular response to reactive oxygen species
PA: Phytanic acid
