# Supplementary material for: Identification of an at-risk subpopulation with high immune infiltration based on the peroxisome pathway and TIM3 in colorectal cancer
Source: BMC Cancer. 2022 Jan 7;22:44. doi: 10.1186/s12885-021-09085-9 (PMC8739708; doi:10.1186/s12885-021-09085-9)
Supplement: Supplementary file 2 — Additional file 2: Figure S1. a The volcano plot shows the enrichment analysis using Hallmark gene sets in the GSE39582 cohort. The red gene sets were enriched in the Per-High group, whereas the blue gene sets were enriched in the Per-Low group. b Correlation between the peroxisome score versus immune score, stromal score, and tumor purity in the GSE39582 cohort. c Boxplots of the immune score, stromal score, and tumor purity from ESTIMATE of Per-High and Per-Low groups in the GSE39582 cohort. d Boxplot of total immune infiltrates (sum of absolute scores across 22 immune cell types) and correlation between peroxisome score and total immune infiltrates of patients in TCGA cohort. For Boxplots, p values in group comparison with Mann-Whitney U-test are shown. For panels B, Pearson’s rho (r) and statistical difference (p) are indicated. **P < 0.01; ****P < 0.0001. Figure S2. Boxplots depict the expression of immune checkpoint genes in the TCGA colorectal dataset. Statistical P values between groups were determined by Mann-Whitney U-test. HM_PEROXISOME: Hallmark Peroxisome gene set. **P < 0.01, ***P < 0.001, ****P < 0.0001. Figure S3. a The log-rank test score at the candidate cut-off across the log-transformed TIM3 gene expression values is plotted. b Kaplan-Meier curves are plotted for the TIM3-High group and TIM3-Low group by the optimal cut-off shown in panel a. Figure S4. GSE39582 group III tumors were highly infiltrated with CD8 T cells and macrophages. a Violin plot showing the total immune infiltrates of CIBERSORTx for each subgroup in the GSE39582 CRC dataset. b Violin plot showing the ESTIMATE tumor purity for each subgroup in the GSE39582 CRC dataset. c Boxplots showing enrichment levels of CD8 T cells and macrophages for each subgroup in the GSE39582 dataset. d, g Boxplots of enrichment level of CD8 T cells (d) and macrophages (g) MSS tumors. e, h Boxplots of enrichment level of CD8 T cells (e) and macrophages (h) for GSE39582 CMS1 and CMS4 tumors. f, i Boxpl [file 12885_2021_9085_MOESM2_ESM.pdf]

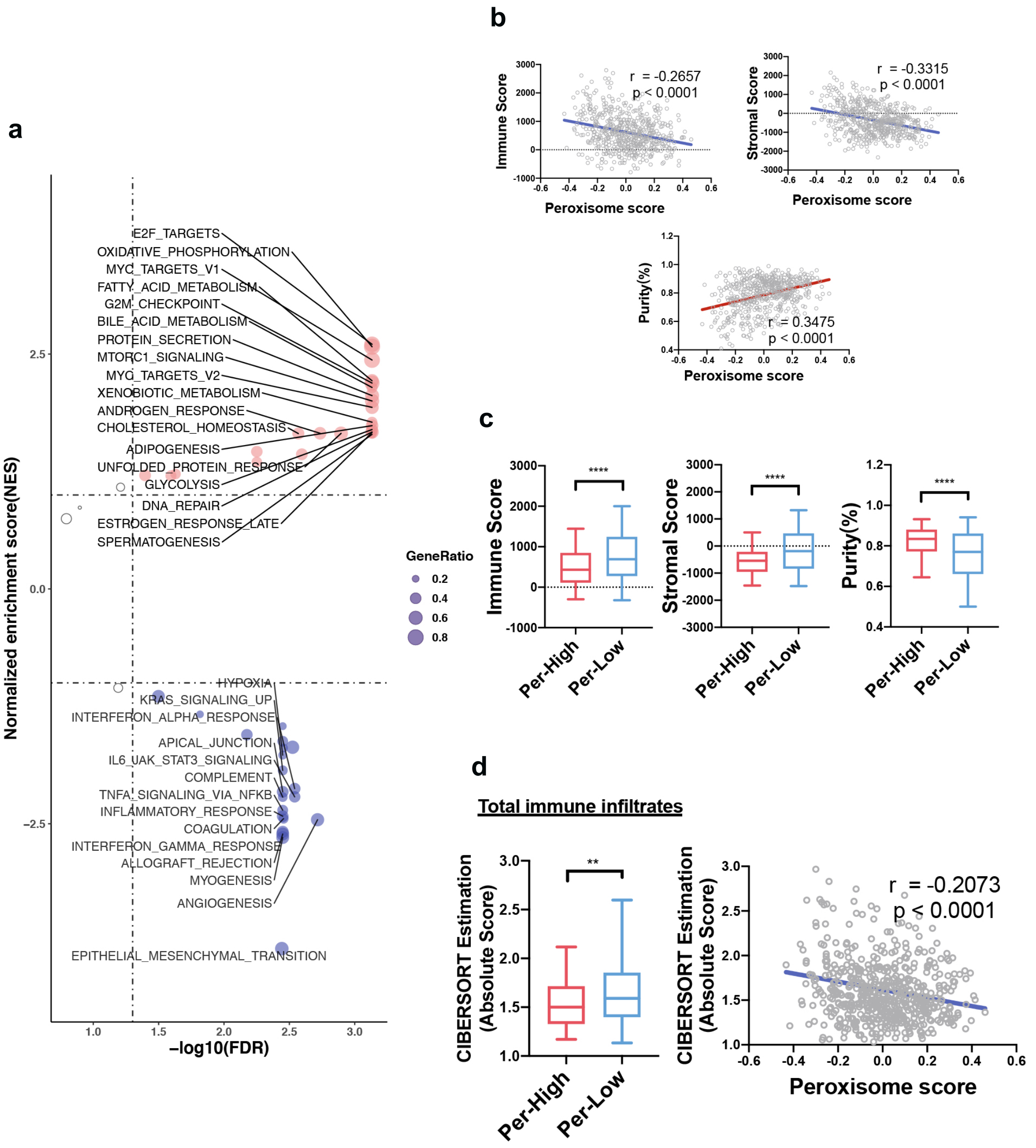

Figure S1. a The volcano plot shows the enrichment analysis using Hallmark gene sets in the GSE39582 cohort. The red gene sets were enriched in the Per-High group, whereas the blue gene sets were enriched in the Per-Low group. **b** Correlation between the peroxisome score versus immune score, stromal score, and tumor purity in the GSE39582 cohort. **c** Boxplots of the immune score, stromal score, and tumor purity

from ESTIMATE of Per-High and Per-Low groups in the GSE39582 cohort. **d** Boxplot of total immune infiltrates (sum of absolute scores across 22 immune cell types) and correlation between peroxisome score and total immune infiltrates of patients in TCGA cohort. For Boxplots, p values in group comparison with Mann-Whitney U-test are shown. For panels B, Pearson's rho (r) and statistical difference (p) are indicated. **P < 0.01; ****P < 0.0001.

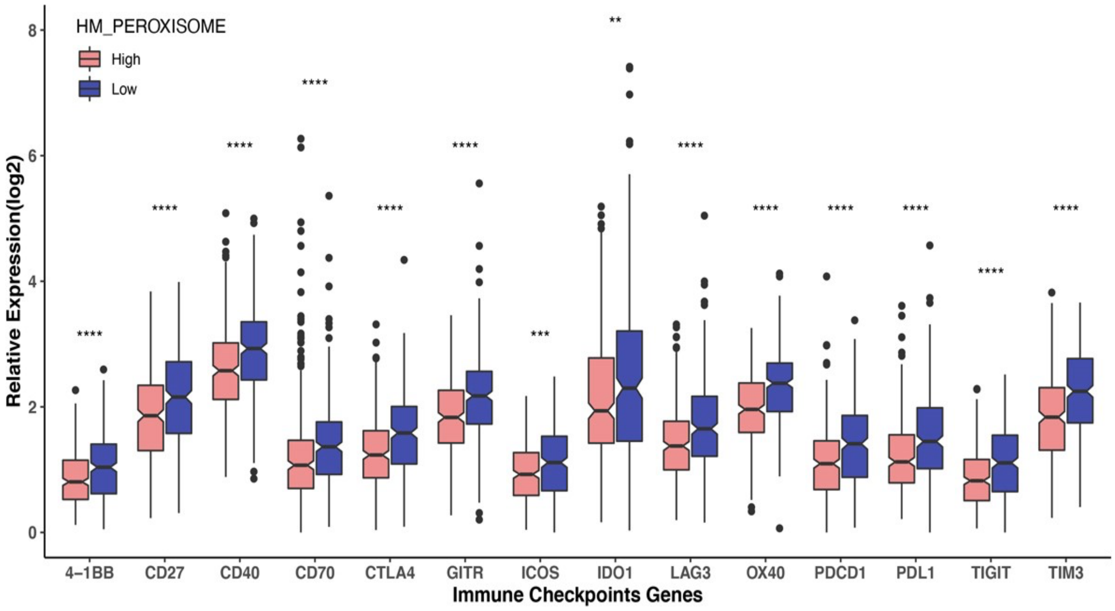

Figure S2. Boxplots depict the expression of immune checkpoint genes in the TCGA colorectal dataset. Statistical P values between groups were determined by Mann-Whitney U-test. HM_PEROXISOME: Hallmark Peroxisome gene set. **P < 0.01, ***P < 0.001, ****P < 0.0001.

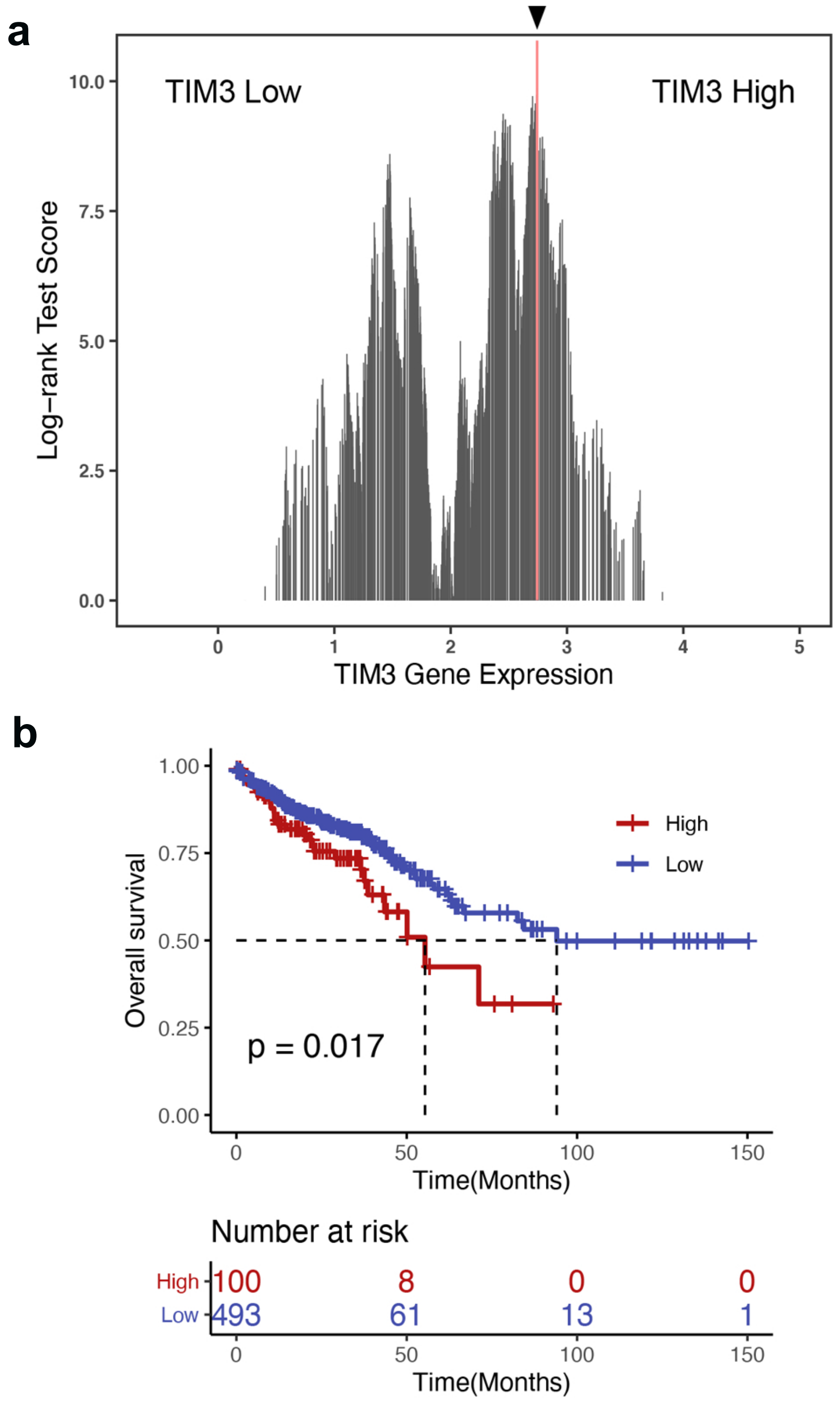

Figure S3. a The log-rank test score at the candidate cut-off across the log-transformed TIM3 gene expression values is plotted. **b** Kaplan-Meier curves are plotted for the TIM3-High group and TIM3-Low group by the optimal cut-off shown in panel a.

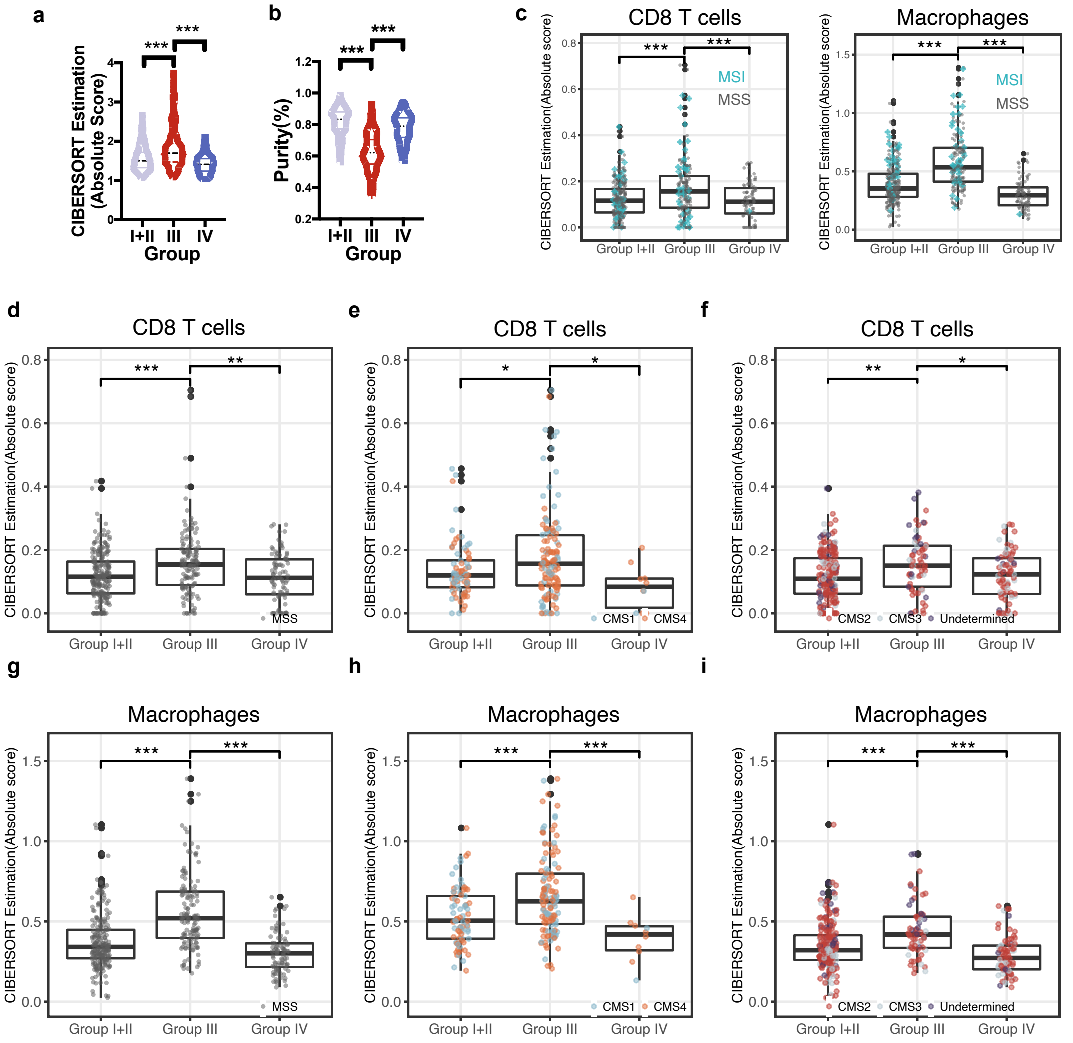

Figure S4. GSE39582 group III tumors were highly infiltrated with CD8 T cells and macrophages. **a** Violin plot showing the total immune infiltrates of CIBERSORTx for each subgroup in the GSE39582 CRC dataset. **b** Violin plot showing the ESTIMATE tumor purity for each subgroup in the GSE39582 CRC dataset. **c** Boxplots showing enrichment levels of CD8 T cells and macrophages for each subgroup in the GSE39582 dataset. **d, g** Boxplots of enrichment level of CD8 T cells (d) and macrophages (g) MSS tumors. **e, h** Boxplots of enrichment level of CD8 T cells (e) and macrophages (h) for GSE39582 CMS1 and CMS4 tumors. **f, i** Boxplots of enrichment level of CD8 T cells

(f) and macrophages (i) for GSE39582 CMS2, CMS3, and indeterminate tumors. *P < 0.05; **P < 0.01; ***P<0.001

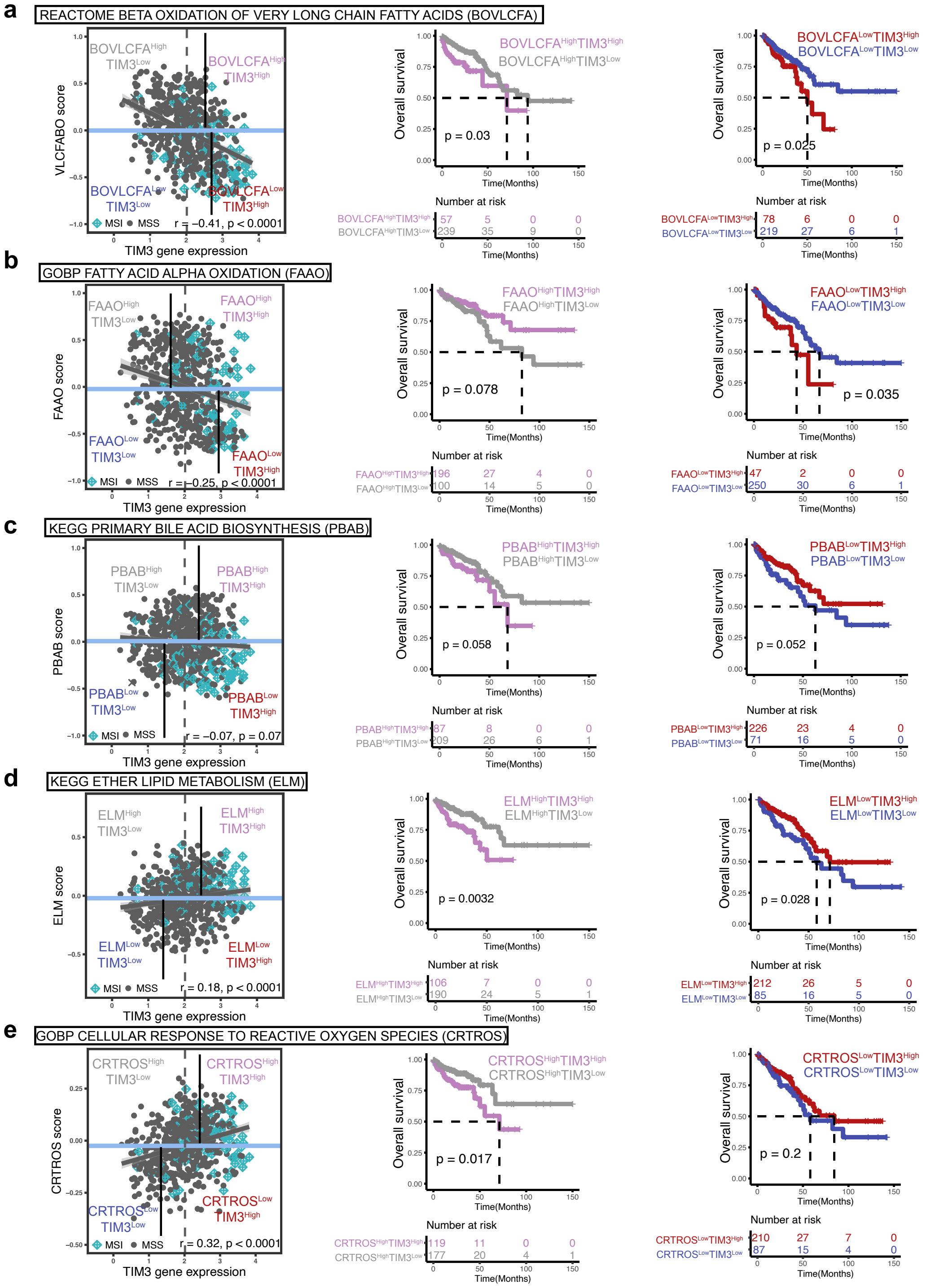

Figure S5. a Scatter plot of BOVLCFA score and log2-transformed TIM3 gene expression values are shown for TCGA cohort. The Pearson's rho (r) and statistical difference (p) are shown in the scatter plot. MSI (blue diamond) and MSS (black circle) status are labeled for CRC patients. The median value of TIM3 expression is indicated

in a gray dashed line. The candidate cut-offs (black lines) for high BOVLCFA and low BOVLCFA groups are shown. Using the median value of BOVLCFA score and these two candidate cut-offs, we separated patients into four different groups labeled. Kaplan-Meier curves show the OS for the optimal cut-off of TIM3 expression in TCGA CRC subgroups with high BOVLCFA score and low BOVLCFA score, respectively. P-value was calculated by the log-rank test and shown for each plot. **b** The same as panel **a**, but for FAAO score. **c** The same as panel **a**, but for PBAB score. **d** The same as panel **a**, but for ELM score. **e** The same as panel **a**, but for CRTROS score. BOVLCFA: beta oxidation of very long chain fatty acids; PBAB: primary bile acid biosynthesis; ELM: ether lipid metabolism; CRTROS: cellular response to reactive oxygen species.
